# The association between Supplemental Nutrition Assistance Program participation and cognitive decline by racial/ethnic groups: a 10‐year longitudinal study

**DOI:** 10.1002/alz70860_097630

**Published:** 2025-12-23

**Authors:** Linlin Da, Zhezheng Jin, Qianhui Xu, Suhang Song

**Affiliations:** ^1^ University of Georgia, Athens, GA, USA; ^2^ Columbia University, New York, NY, USA; ^3^ New York University, New York, NY, USA

## Abstract

**Background:**

The Supplemental Nutrition Assistance Program (SNAP) plays a significant role in addressing food insecurity, but its potential impact on cognitive decline in older adults remains largely unexplored. Few studies have examined the relationship between SNAP participation and cognitive health, with limited focus on racial/ethnic disparities and specific cognitive domains. This study investigates the association between SNAP participation and cognitive decline and assesses whether such association differs across racial/ethnic groups and cognitive domains.

**Method:**

The analytic sample (*n* = 2,347) includes participants in the Health and Retirement Study who were enrolled in SNAP in 2010. We defined SNAP users as eligible if their household income was at or below 130% of the federal poverty threshold at the time of the interview. Cognition was assessed biennially from 2010 through 2020 using a telephone‐based cognitive status interview or web‐based interviews. Linear mixed regression models were used to estimate the associations between SNAP participation and cognitive decline by racial/ethnic groups.

**Result:**

This study found that SNAP participation was associated with a slower rate of cognitive decline in global cognition (β = 0.09, 95% CI: 0.05, 0.14, *p* < 0.001), memory (β = 0.07, 95% CI: 0.03, 0.11, *p* < 0.001), and executive function (β = 0.03, 95% CI: 0.01, 0.04, *p* = 0.004). When stratified by racial/ethnic groups, a slightly faster decline in global cognition and memory was observed among non‐Hispanic Black (global cognition: β = ‐0.12, 95% CI: ‐0.22, ‐0.01, *p* = 0.04; memory: β = ‐0.08, 95% CI: ‐0.17, 0.01, *p* = 0.09) and Hispanic (global cognition: β = ‐0.12, 95% CI: ‐0.23, ‐0.01, *p* = 0.03; memory: β = ‐0.10, 95% CI: ‐0.19, ‐0.00, *p* = 0.05) compared to non‐Hispanic White. No significant racial/ethnic differences were observed for executive function.

**Conclusion:**

Our findings suggest that SNAP participation may provide a protective effect on slowing cognitive decline, and such an effect differs by racial/ethnic groups. These results underscore the significance of nutrition assistance programs in supporting cognitive health in older adults. Future studies are warranted to further explore the underlying mechanisms between SNAP and cognitive health by racial/ethnic groups.

